# Daily consumption of wild olive (acebuche) oil reduces blood pressure and ameliorates endothelial dysfunction and vascular remodelling in rats with NG-nitro-L-arginine methyl ester-induced hypertension

**DOI:** 10.1017/S0007114522000034

**Published:** 2022-10-14

**Authors:** Claudia Reyes-Goya, Álvaro Santana-Garrido, Gema Aguilar-Espejo, M. Carmen Pérez-Camino, Alfonso Mate, Carmen M. Vázquez

**Affiliations:** 1 Departamento de Fisiología, Facultad de Farmacia, Universidad de Sevilla, E-41012 Sevilla, Spain; 2 Epidemiología Clínica y Riesgo Cardiovascular, Instituto de Biomedicina de Sevilla (IBIS), Hospital Universitario Virgen del Rocío/Consejo Superior de Investigaciones Científicas, Universidad de Sevilla, E-41013 Sevilla, Spain; 3 Departamento de Caracterización y Calidad de lípidos, Instituto de la Grasa-CSIC, E-41013 Sevilla, Spain

**Keywords:** Acebuche, Aorta, Arterial hypertension, Endothelial dysfunction, NG-nitro-l-arginine methyl ester, Wild olive oil

## Abstract

Despite numerous reports on the beneficial effects of olive oil in the cardiovascular context, very little is known about the olive tree’s wild counterpart (*Olea europaea*, L. var. *sylvestris*), commonly known as *acebuche* (ACE) in Spain. The aim of this study was to analyse the possible beneficial effects of an extra virgin ACE oil on vascular function in a rodent model of arterial hypertension (AH) induced by N^G^-nitro-l-arginine methyl ester (L-NAME). Four experimental groups of male Wistar rats were studied: (1) normotensive rats (Control group); (2) normotensive rats fed a commercial diet supplemented with 15 % (w/w) ACE oil (Acebuche group); (3) rats made hypertensive following administration of L-NAME (L-NAME group); and (4) rats treated with L-NAME and simultaneously supplemented with 15 % ACE oil (LN + ACE group). All treatments were maintained for 12 weeks. Besides a significant blood pressure (BP)-lowering effect, the ACE oil-enriched diet counteracted the alterations found in aortas from hypertensive rats in terms of morphology and responsiveness to vasoactive mediators. In addition, a decrease in hypertension-related fibrotic and oxidative stress processes was observed in L-NAME-treated rats subjected to ACE oil supplement. Therefore, using a model of AH via nitric oxide depletion, here we demonstrate the beneficial effects of a wild olive oil based upon its vasodilator, antihypertensive, antioxidant, antihypertrophic and antifibrotic properties. We postulate that regular inclusion of ACE oil in the diet can alleviate the vascular remodelling and endothelial dysfunction processes typically found in AH, thus resulting in a significant reduction of BP.

The traditional Mediterranean diet is generally based on grains, cereals, vegetables, nuts, fruits, meat, fish, moderate consumption of milk, low-to-moderate wine consumption and use of olive oil as the main source of fat. Recent meta-analysis of observational studies showed that Mediterranean diet can reduce the risk of CVD^(1)^. Moreover, beneficial effects of this diet have been associated with lower risk of hypertension, cancer, Alzheimer’s disease, diabetes, the metabolic syndrome, obesity, hyperglycaemia and hyperlipidaemia^(2–9)^.

Olive oil has been recognised as the golden standard or main element in the diet in healthy nutrition. In addition to oleic acid, minor constituents such as triterpenes, polyphenols, tocopherols and sterols add bioactive properties to virgin olive oil (VOO)^(10–13)^. Triterpenic compounds (e.g., maslinic acid, oleanolic acid, erythrodiol and uvaol) have shown promise against vascular dysfunction and prevention of CVD progression both in humans and in rodent models^(14,15)^. Beneficial effects of polyphenol-enriched diets have also been reported in the setting of diabetes, the metabolic syndrome (excessive body weight/adiposity, dyslipidaemia, hypertension and hyperglycaemia/insulin resistance), cancer, and cardiovascular and degenerative diseases, based on the antioxidant, anti-inflammatory and antiangiogenic effects of these compounds^(16–20)^. Likewise, the importance of tocopherols and plant sterols in human health lies in their antioxidant, hypocholesterolaemic and anti-inflammatory capacity^(12,13,21)^.

*Acebuche* (ACE), or wild olive tree (*Olea europaea*, L. var. *sylvestris*), is an ancient variety of olive tree mainly used to obtain olives and olive oil. ACE is distributed in Mediterranean countries^(22,23)^, with a high presence in the Iberian Peninsula and specifically in Southern Spain^(24)^. In contrast to the extensive literature covering the health effects of cultivated olive oil, reports on the chemical composition and/or therapeutic effect of wild olive (ACE) oil are very scarce. Olive tree ancestors seem to have lower allergenicity than the widely distributed cultivated lineages, and preliminary studies found a higher amount of tocopherols and sterols in ACE oil compared with standard extra virgin olive oils (EVOO)^(25,26)^. We have recently reported the presence of a higher proportion of triterpene acids/alcohols and secoiridoid polyphenols in ACE oil than in EVOO, which might explain its antioxidant and retinoprotective potential in the eye of hypertensive mice^(27)^.

Arterial hypertension (AH) has long been recognised as one of the most powerful risk factors for cardiovascular events, including myocardial infarction, stroke, heart failure and chronic renal failure^(28)^. In this sense, and besides the wide range of antihypertensive drugs, promoting a healthy lifestyle that includes nutraceutical foods such as ACE oil might help control blood pressure (BP)^(27)^. In the hypertensive setting, many reports support the notion that NADPH oxidase has a pivotal role in reactive oxygen species production, specially superoxide anion (O_2_^•^-) and peroxide hydrogen (H_2_O_2_), with subsequent organ damage^(29)^. Seven isoforms (NOX1–5 and Duox1–2) of the catalytic subunit of this enzyme complex are differentially expressed in the body^(30)^, of which isoforms NOX1, 2, 4 and 5 are predominant in vascular cells^(31)^.

The aim of this work was to explore the beneficial effects of ACE oil to prevent AH-associated vascular dysfunction. To this purpose, rats made hypertensive via administration of N^G^-nitro-l-arginine methyl ester (L-NAME, which constitutes a validated model for studying hypertension-related endothelial dysfunction and vascular wall remodelling^(32)^) were simultaneously administered a diet enriched with 15 % (w/w) of ACE oil. Studies of vascular reactivity in the aorta were complemented with morphometric measurements, Sirius Red staining and immunolocalisation/immunoblotting of transforming growth factor beta 1 (TGF-*β*1), in order to determine the degree of fibrosis of this vessel in the different experimental conditions. In addition, NADPH oxidase activity/expression, superoxide anion production in the aortic wall, relative quantification of total endothelial nitric oxide synthase (eNOS) enzyme and the ratio p-eNOS Ser^1177^/ p-eNOS Thr^495^, as well as the expression of antioxidant enzymes glutathione peroxidase (GSH-Px1/2), glutathione reductase (GSH-Red), and superoxide dismutase (SOD-1), were measured in aortas from normotensive and hypertensive animals to evaluate the antioxidant capacity of ACE oil. Systemic parameters were also tested following dietary supplementation with wild olive oil, including the activity of antioxidant enzymes in peripheral blood, and plasma lipid profile, NO and urea levels as an estimate of renal function.

## Materials and methods

### Animals and experimental design

The study was carried out in accordance with the European Union (EU Directive 2010/63/EU) and the National (RD 53/2013) guidelines for the care and use of laboratory animals and was approved by the competent experimentation ethics committee at the University of Sevilla, Spain, where the animal work took place (approval reference # 22/10/2018/148, issued by Junta de Andalucía, Dirección General de la Producción Agraria y Ganadera). Male Wistar rats (8–10 weeks old, average initial weight 273 (sem 6) g) were obtained from the Servicio de Producción y Experimentación Animal at the University of Seville (Spain) and maintained under standard conditions in transparent polycarbonate open-top cages (23 (s em 1)°C, 12 h/12 h light/dark cycles). Four groups of six animals each (two animals per cage) were randomly established: nonhypertensive groups, (i) Control group (free access to tap water and fed a standard diet (AIN-93M)); (ii) rats fed AIN-93M diet supplemented with 15 % (w/w) ACE oil (Acebuche group); and induced hypertension groups: (iii) rats fed AIN-93M diet and treated with 20 mg L-NAME/kg body weight/d, dissolved in drinking water (L-NAME group); and (iv) rats treated with both L-NAME and 15 % (w/w) ACE oil-enriched AIN-93M diet (LN + ACE group). Carbohydrate content was corrected, when necessary, to render all diets isoenergetic. In order to ensure correct dosage, the concentrations of L-NAME were adjusted weekly according to the animals’ body weight and water intake. The controlled feeding/treatment period lasted 12 weeks. No differences in food or water intake were observed among the different groups of animals during this time.

ACE oil was obtained and processed to obtain its chemical composition following the previous protocols described in Santana-Garrido^(27)^. Briefly, to prepare the animal feed, all components from AIN-93M: maize starch, casein, sucrose, soyabean oil (no additives), fibre, mineral mix (AIN-93G-MX),vitamin mix (AIN-93-VX), l-cystine, choline bitartrate, tert-Butylhydroquinone and ACE oil were mixed in corresponding quantities as appropriate, ensuring a homogenate oil spread in the powder, up to a final 15 % (w/w) of ACE olive oil. This powder–oil mixture was used to create feed pellets, which were kept fresh and light protected until use.

### Blood pressure and tissue preparations

BP by tail-cuff occlusion and body weight were continuously monitored throughout the experimental period as previously described^(33)^. Upon treatment completion, animals were injected intraperitoneally with 75 mg/kg ketamine + 10 mg/kg diazepam. Blood samples were collected by cardiac puncture into tubes containing lithium heparin. The intact thoracic aorta was removed and washed in ice-cold 0·9 % saline solution. The superior portion (including the aortic arch) was immediately frozen in liquid N_2_ and stored at –80°C until use for measuring NADPH oxidase activity and for protein/gene expression analyses; the rest was employed for functional determinations (vascular reactivity), histomorphometric studies, dihydroethidium (DHE) staining and immunohistochemistry, as detailed below. All animals were routinely killed between 09.00 and 10.00 hours to minimise diurnal variations.

### Plasma lipid profile and urea levels

Plasma was separated by centrifugation (2500 ***g***, 15 min) and stored at –80°C until use. The concentrations of total cholesterol, TAG, total lipids, phospholipids, LDL-cholesterol, HDL-cholesterol and urea were measured using commercial kits (Spinreact).

### Vascular reactivity

Vascular function was analysed as previously reported^(34)^ using a Panlab compact organ bath and a PowerLab® 8/30 data acquisition system coupled to appropriate transducers and controlled by LabChart^TM^ software (ADInstruments). Endothelium-intact aortic rings were contracted with 10^−9^–3 × 10^−5^ mol/l phenylephrine and relaxed with 10^−9^–3 × 10^−5^ mol/l acetylcholine (ACh) or 10^−10^–3 × 10^−6^ mol/l sodium nitroprusside.

### Histomorphometric studies

Slices from thoracic aortas were haematoxylin–eosin stained and photographed with an Olympus BX41 microscope (Olympus Iberia S.A.U). The area and thickness of tunica media and the lumen area were calculated using ImageJ software (Version 2.0.0-rc-69/1·52p). Additional slices were also stained with 0·1 % picro-Sirius Red (Sigma-Aldrich Inc) for fibrosis detection. Random aortic rings were captured and processed with a high-resolution video camera (DF-WX710, Sony Europe B.V) connected to a light microscope (Nikon Eclipse 50i) using the 20× objective and a green optical filter (IF 550). The area occupied by collagen was measured using a computerised image analysis system (Fibrosis HRR, Master Diagnostica), as previously described^(35)^. The values obtained for fibrous tissue were expressed in square micrometers.

### Measurement of superoxide anion and nitric oxide levels, and NADPH oxidase activity

The superoxide-sensitive fluorescent dye DHE (MedChemExpress, HY-D0079) was used to estimate the level of superoxide anion (O_2_^•^-) as previously reported^(36)^. Thoracic aortas were embedded in Tissue-Tek® 4583 O.C.T. compound (Sakura) and frozen sections were cut into 10 µm thick sections with a cryostat (Leica CM1510 S, Leica Biosystems Nussloch GmbH). Sections were thawed, incubated with DHE (10 mmol/l) at 37°C for 10 min and with 4’,6-diamidino-2-phenylindole Fluoromount-G® (SouthernBiotech 0100-20). Preincubation with 100 U/ml polyethylene glycol-conjugated SOD (Sigma-Aldrich, S9549, Merck Life Science S.L.U.) for 10 min at 37°C was carried out to confirm the specificity of staining. All sections were examined under an Olympus BX61 fluorescence microscope and photographed with an Olympus DP73 colour digital camera (Olympus Iberia S.A.U.). O_2_^•^- production was estimated from the ratio ethidium/4’,6-diamidino-2-phenylindole fluorescence signal using ImageJ software. In turn, aorta homogenates were used to determine nitric oxide levels by a Nitrite Assay Kit (Sigma-Aldrich MAK367, Merck Life Science S.L.U.), as well as NADPH oxidase enzyme activity by the lucigenin-enhanced chemiluminescence, as previously reported^(37)^.

### Immunohistochemistry

Immunohistochemistry was carried out on paraffin-embedded sections using mouse monoclonal anti-TGF-*β*1 (3C11, Santa Cruz Biotechnology; 1:50 dilution); an adequate anti-mouse secondary antibody was then used following manufacturer’s recommendations. Signal was revealed with standard immunohistochemistry methods and SIGMAFAST™ DAB Tablets (Sigma-Aldrich, D0426, Merck Life Science S.L.U.) as a chromogen. Slides were co-stained with haematoxylin and photographed with an Olympus BX41 microscope (Olympus Iberia S.A.U.).

### Western blotting

Aorta tissue was homogenised in radioimmunoprecipitation assay buffer with a micro-pestle motor-driven tissue homogeniser (Heidolph Instruments). After centrifugation at 2000 × ***g*** for 10 min at 4°C, the supernatant was stored at −80°C until use. The Bradford method (Bio-Rad Protein Assay, Bio-Rad Laboratories, S.A.) was used for determining protein concentration^(38)^. Following SDS-PAGE, proteins were transferred to nitrocellulose membrane and incubated with relevant primary antibodies, namely mouse monoclonal anti-TGF-*β*1 (3C11, Santa Cruz Biotechnology; 1:3000 dilution), mouse monoclonal anti-NOS3 (A-9) (Santa Cruz Biotechnology; 1:2000 dilution); purified mouse anti-eNOS (pS1177) (BD Transduction Laboratories, 1:2000 dilution); mouse monoclonal anti-p-NOS3 (pt495·33) (Santa Cruz Biotechnology; 1:2000 dilution); rabbit polyclonal anti-GSH-Px1/2 (Santa Cruz Biotechnology; 1:1000 dilution); rabbit polyclonal anti-GSH-Red (Santa Cruz Biotechnology; 1:5000 dilution); and mouse monoclonal anti-SOD-1 (Santa Cruz Biotechnology; 1:1000 dilution). Mouse monoclonal anti-*β*-actin (Santa Cruz Biotechnology; 1:20 000 dilution) was also used for protein loading control. Anti-mouse or anti-rabbit secondary antibody, as appropriate, were used following manufacturer’s recommendations, and signals were revealed with Amersham ECL Western Blotting Detection Kit (RPN2232) and measured with an Amersham Imager 600 (Cytiva Europe GmbH).

### Real-time PCR

Aortic RNA was isolated by the TRIzol® RNA isolation method (Thermo Fisher Scientific) and retrotranscribed as reported elsewhere^(27)^. Specific primers (listed in Table 1) were used for the amplification of gene products in a CFX96 real-time PCR system (Bio-Rad). As an internal control housekeeping gene, glyceraldehyde-3-phosphate dehydrogenase was used to quantify the relative changes in mRNA expression in each corresponding group, using the 2^−ΔΔCt^ method^(39)^.

**Table 1.** Primers used for real-time PCR experiments

	Forward (5’-3’)	Reverse (5’-3’)
NOX1	TTCACCAATTCCCAGGATTGAAGTGGATGGTC	GACCTGTCACGATGTCAGTGGCCTTGTCAA
NOX2	CCCTTTGGTACAGCCAGTGAAGAT	CAATCCCAGCTCCCACTAACATCA
NOX4	TTGCTTTTGTATCTTC	CTTACCTTCGTCACAG
GAPDH	GCCAAAAGGGTCATCATCTCCGC	GGATGACCTTGCCCACAGCCTTG

GAPDH, glyceraldehyde-3-phosphate dehydrogenase.

### Enzyme activity measurements

The activities of GSH-Px1/2, GSH-Red and SOD-1 enzymes were assayed using commercial kits (Randox Laboratories) according to previously reported protocols. Results were expressed relative to Hb content in the corresponding blood samples^(40)^.

### Statistics

Sample size was estimated from previous work in our laboratory using the L-NAME model of AH and assuming a 25 % difference in the mean, with a statistical power of 90 % and a 5 % significance level^(41)^. Data were analysed with GraphPad Prism version 5.01 (GraphPad Software) and expressed as mean values with their standard errors of the mean. ANOVA with Tukey’s post-hoc test was used for comparisons between all four experimental groups.

## Results

### General characteristics of animals

**Fig. 1.**
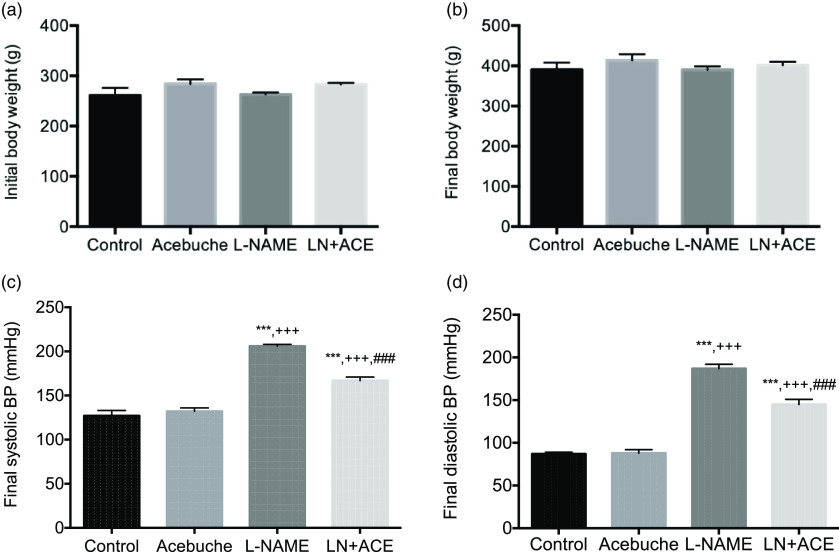
(a) Initial and (b) final body weight, and final (c) systolic and (d) diastolic blood pressure (BP) in the different experimental groups. Values are expressed as mean values with their standard errors of the mean of six animals per group. ***P < 0·001 *v*. Control group; ^+++^ *P* < 0·001 *v*. Acebuche group; ^###^ *P* < 0·001 *v*. L-NAME group.

No differences in final body weight were observed among the experimental groups at the end of the 12-week experimental period (Fig. 1(a) and (b)). As expected, arterial BP was elevated in rats treated with L-NAME alone. Notably, dietary supplementation with ACE oil decreased both systolic and diastolic BP in hypertensive rats (LN + ACE group), although the values remained higher than those of normotensive rats. No significant differences were observed in final BP levels between Control and Acebuche groups (Fig. 1(c) and (d)).

### Plasma biochemical profile

No changes in total lipid content, total cholesterol and phospholipids were observed among the different experimental groups. On the other hand, the LN + ACE group showed a significant rise in plasma HDL-cholesterol and a parallel decrease in LDL-cholesterol. The concentration of TAG increased in hypertensive animals when compared with the Control group, an alteration that could be prevented by dietary supplementation with ACE oil (Table 2).

**Table 2.** Plasma lipid profile and urea levels (Mean values with their standard errors of the mean)

	Control		Acebuche		L-NAME		LN + ACE	
	Mean	sem	Mean	sem	Mean	sem	Mean	sem
Total cholesterol (mg/dl)	49·2	0·5	62·2	1·9	52·1	6·7	56·5	3·3
TAG (mg/dl)	56·5	1·1	61·3	2·9	78	1·3***,††	66·4	3·2*,‡
LDL-cholesterol (mg/dl)	21·4	2·3	15·9	1·4	17·8	1·7	11·3	0·84**,‡
HDL-cholesterol (mg/dl)	22·3	1	36·0	2*	27·4	2·3	38·1	3·2**,‡
Total lipids (mg/dl)	353·6	14	431·1	30·8	389·6	16·9	417	17·5
Phospholipids (mg/dl)	71·2	11·2	58·1	1·4	46·3	4·2	75·7	8·3
Urea (mg/dl)	4·3	0·2	6·2	2·0	17·5	0·7***,†	9·0	1·8‡

Results correspond to mean values with their standard errors of the mean of four animals per group.

*P < 0·05, **P < 0·01, ***P < 0·001 *v.* Control group; †*P* < 0·01, ††*P* < 0·001 *v.* Acebuche group; ‡*P* < 0·05 *v.* L-NAME group.

Interestingly, readings of plasma urea levels revealed a considerable elevation of this parameter in rats treated with L-NAME, while administration of our ACE oil-enriched diet for 12 weeks restored this marker of kidney function to normal (Table 2).

### Acebuche oil-enriched diet alleviates vascular dysfunction and improves nitric oxide bioavailability

**Fig. 2.**
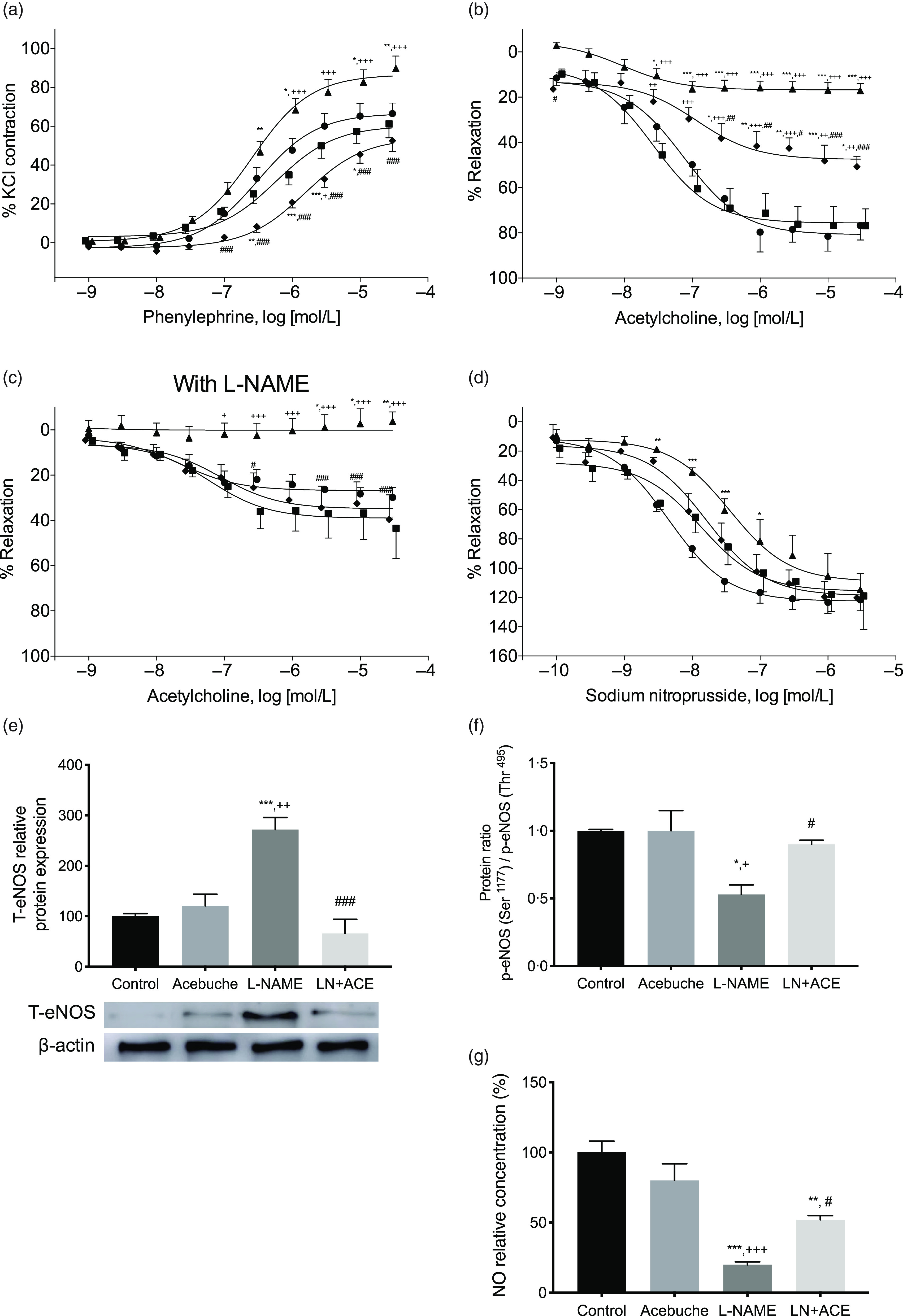
Dose–response curves in aortic rings corresponding to (a) vasoconstriction mediated by phenylephrine (10^−9^–3 × 10^−5^ mol/l); (b) acetylcholine-mediated (10^−9^–3 × 10^−5^ mol/l) vasodilation; (c) same experiment as in (b) previous acute incubation with L-NAME (10^−4^ mol/l) for 30 min; (d) vasorelaxation response to sodium nitroprusside (10^−10^–3 × 10^−6^ mol/l). The results are expressed as relative percentages of the maximum contraction induced by 60 mmol/L KCl (a), or the contraction induced by a submaximal dose of phenylephrine (b)–(d); (e) total eNOS protein expression; (f) ratio p-eNOS (Ser^1177^) to p-eNOS (Thr^495^); (G) NO levels in aorta homogenates of rats from the different experimental groups. Values are expressed as mean values with their standard errors of the mean of six animals per group. *P < 0·05, **P < 0·01, ***P < 0·001 *v*. Control group; ^+^ *P* < 0·05, ^++^ *P* < 0·01, ^+++^ *P* < 0·001 *v*. Acebuche group; ^#^ *P* < 0·05, ^##^ *P* < 0·01, ^###^ *P* < 0·001 *v*. L-NAME group. (a–d) 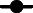, Control; 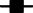, Acebuche; 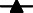, L-NAME; 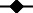, LN + ACE

Vascular reactivity studies showed an increase in the vasoconstrictor response to phenylephrine in aortic rings from hypertensive rats when compared with Control and Acebuche groups, an alteration that was blocked in hypertensive animals receiving ACE oil (Fig. 2(a)). Thus, a significant decrease in E_max_ and pEC_50_ values was observed in LN + ACE group in comparison with L-NAME group (Table 3).

**Table 3.** *E*_max_ and pEC_50_ values from vascular reactivity experiments (Mean values with their standard errors of the mean)

Agonists	Control				Acebuche				L-NAME				LN + ACE			
	*E*_max_ (%)		pEC_50_ (-log mol/l)		*E*_max_ (%)		pEC_50_ (-log mol/l)		*E*_max_ (%)		pEC_50_ (-log mol/l)		*E*_max_ (%)		pEC_50_ (-log mol/l)	
	Mean	sem	Mean	sem	Mean	sem	Mean	sem	Mean	sem	Mean	sem	Mean	sem	Mean	sem
PE	67	3	6·5	0·1	60	3	6·2	0·14	87	3**,†	6·6	0·09	54	3*,‡	5·8	0·1**,‡
ACh	81	3	7·1	0·14	76	3	7·6	0·18	17	1**,†	8	0·28*	48	3**,†,‡	6·9	0·24‡
ACh + L-NAME	27	2	7·6	0·29	39	4	7·2	0·39	5	2**,†	5·2	2	35	4‡	7·1	0·36
SNP	123	3	8·3	0·09	116	7	7·9	0·21	109	6	7·4	0·16*	119	5	7·8	0·13

PE, phenylephrine; ACh, acetylcholine; L-NAME, N^G^-nitro-L-arginine methyl ester; SNP, sodium nitroprusside.

Results correspond to mean values with their standard errors of the means of six animals per group. E_max_: maximal effect (vasoconstriction or vasodilation, where applicable) induced by the corresponding agonist. pEC_50_ = -log EC_50_; EC_50_ is the concentration of the corresponding agonist that induces 50 % of its maximal effect.

*P < 0·05, **P < 0·001 *v*. Control group; †*P* < 0·001 *v*. Acebuche group; ‡*P* < 0·001 *v*. L-NAME group.

The maximal vasorelaxation response (E_max_) to ACh was extremely weakened in aortas from hypertensive animals with respect to the values reached in the Control and Acebuche groups. Simultaneous administration with ACE oil resulted in a significant recovery of this endothelium-dependent vasodilation (Fig. 2(b) and Table 3). Interestingly, when we tested the remaining ability of the vessels to respond to ACh in the presence of L-NAME (10^−4^ mol/l, 30 min preincubation), hypertensive rats treated with ACE oil behaved the same as the Control and Acebuche groups, whereas aortic rings from the L-NAME group showed negligible E_max_ under these conditions (Fig. 2(c) and Table 3). No changes were observed in this case regarding EC_50_ parameter.

Results concerning endothelium-independent vasorelaxation (i.e. vasodilation measured in response to nitric oxide donor sodium nitroprusside) did not reveal significant changes in E_max_ among the four experimental groups, although the curve shifted slightly to the right in hypertensive, L-NAME-treated rats, which presented with higher EC_50_ (i.e. lower pEC_50_) values (Fig. 2(d) and Table 3). As mentioned, no differences were found between Control and Acebuche groups in any of the vascular reactivity experiments.

Additional experiments on the expression of eNOS in aorta homogenates demonstrated an up-regulation of this synthase enzyme in the L-NAME group that was blocked when these hypertensive rats received dietary supplements with ACE oil (Fig. 2(e)). On the other hand, when we calculated the ratio p-eNOS (Ser^1177^)/p-eNOS (Thr^495^), a deactivation of eNOS subsequent to enhanced phosphorylation at the latter residue (which reflects an inhibitory phosphorylation) was found in the L-NAME group; this alteration was also absent in the LN + ACE group (Fig. 2(f)). It is noteworthy that alterations in the expression/activation of eNOS in hypertensive animals were accompanied by lower levels of NO, and that the bioavailability of this vasodilator molecule in the aorta was significantly recovered after administration of ACE oil (Fig. 2(g)).

### Acebuche oil consumption can improve vascular remodelling

**Fig. 3.**
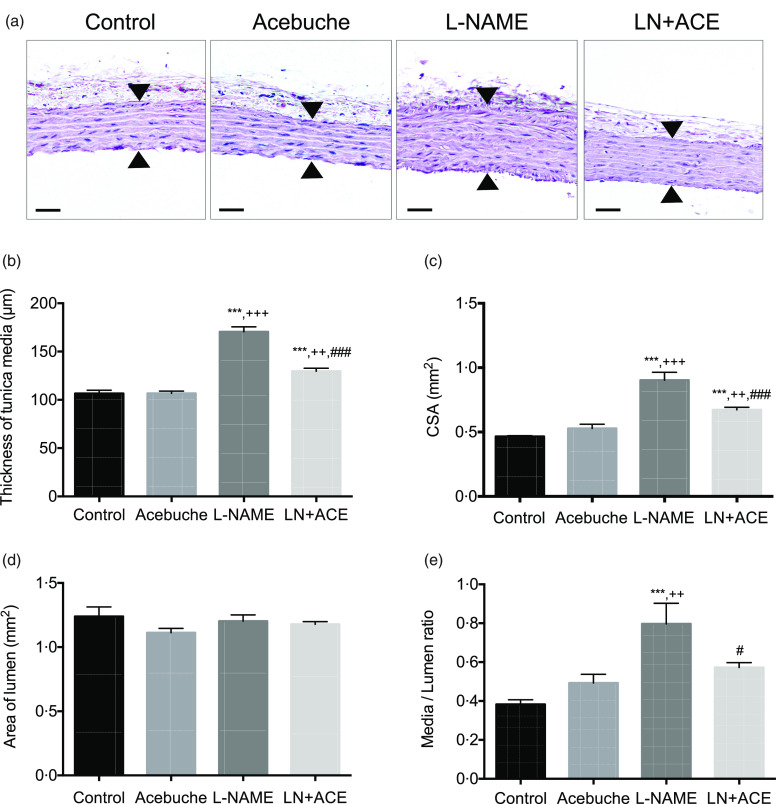
(a) Haematoxylin–eosin staining of aorta rings; (b) thickness of tunica media; (c) area of tunica media; (d) area of lumen and (e) media/lumen ratio in the different experimental groups. Values are expressed as mean values with their standard errors of the mean of six animals per group. ***P < 0·001 *v*. Control group; ^++^ *P* < 0·01, ^+++^ *P* < 0·001 *v*. Acebuche group; ^#^ *P* < 0·05, ^###^ *P* < 0·001 *v*. L-NAME group. Scale bar: 50 µm.

Morphometric studies showed a remarkable thickening of the tunica media in L-NAME-treated animals, which was partly reversed in hypertensive animals subjected to ACE oil supplementation (Fig. 3(a) and (b)); a similar pattern applies to the cross-sectional area (Fig. 3(c)). Since the luminal area did not differ among experimental groups (Fig. 3(d)), a significant rise in the media/lumen ratio was found in L-NAME-treated rats (Fig. 3(e)); this suggests the presence of vascular remodelling in these hypertensive animals, which was abolished by simultaneous intake of ACE oil. The administration of ACE oil to normotensive animals (Acebuche group) had no effect on the aorta morphology.

### Administration of acebuche oil reduces aorta fibrosis

**Fig. 4.**
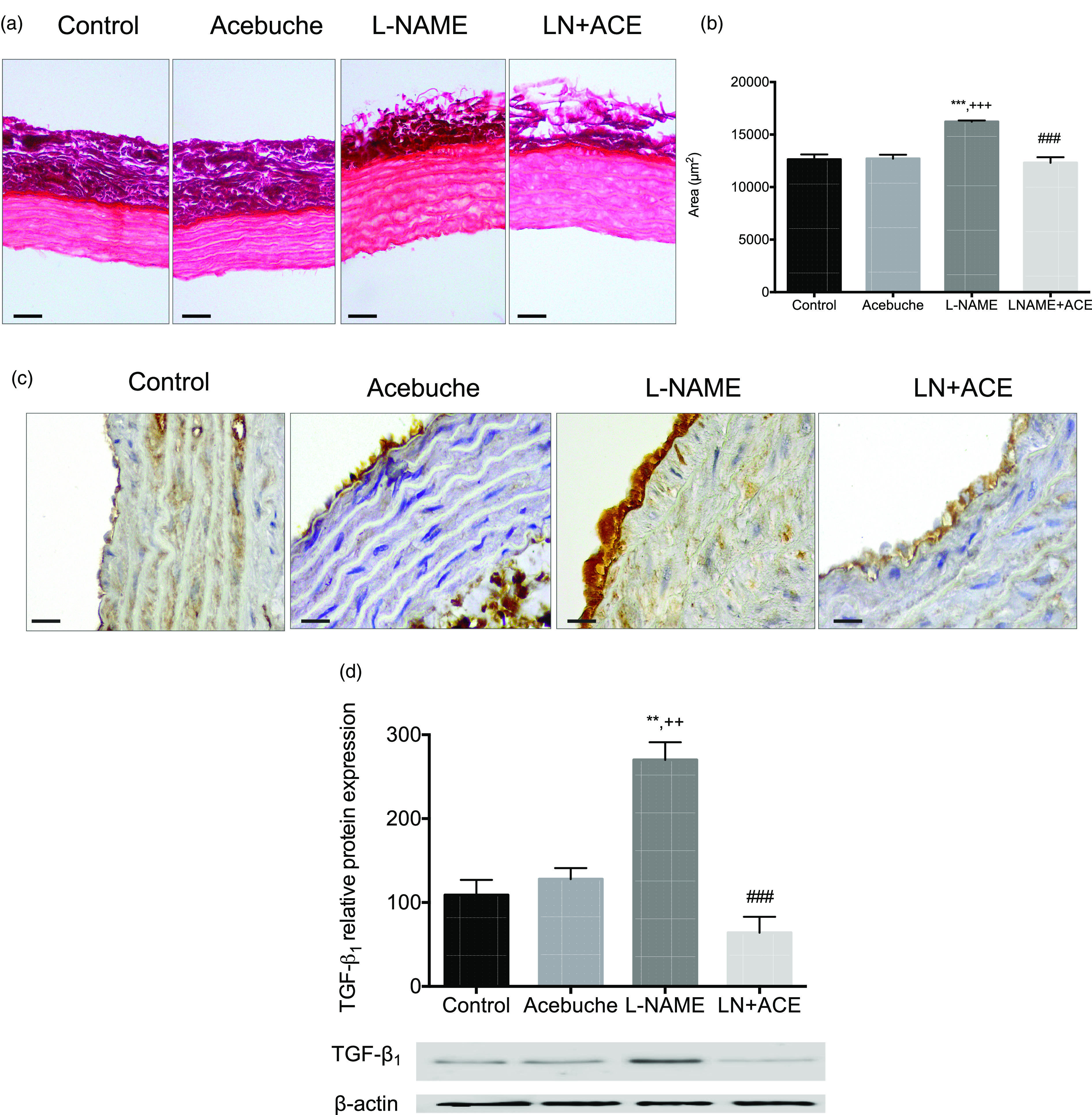
(a) Sirius Red staining of aortas (scale bar: 50 µm); (b) morphometric analysis of areas depicted in (a); (c) TGF-*β*1 immunohistochemistry (scale bar: 20 µm); (d) TGF-*β*1 protein expression in the different experimental groups. Values are expressed as mean values with their standard errors of the mean of six animals per group. **P < 0·01, ***P < 0·001 *v*. Control group; ^++^ *P* < 0·01, ^+++^ *P* < 0·001 *v*. Acebuche group; ^###^ *P* < 0·001 *v*. L-NAME group.

Fibrosis studies in aorta sections showed an increase in the content of total collagen in the tunica media of vessels from hypertensive rats, which disappeared when these animals were fed the ACE oil-containing diet (Fig. 4(a) and (b)). No significant differences were observed in collagen deposition between Control and Acebuche groups.

Immunohistochemistry staining exhibited the greatest expression of TGF-*β*1 in the tunica intima of the L-NAME group, while aortas from the LN + ACE group had a similar appearance to those from normotensive animals (Fig. 4(c)). These results correlated with increased protein expression of TGF-*β*1, estimated by Western blotting, in thoracic aorta homogenates from L-NAME-treated rats, an alteration that was also reversed via ACE oil administration (Fig. 4(d)).

### Reduction of superoxide anion levels and NADPH oxidase activity/expression following acebuche oil consumption

**Fig. 5.**
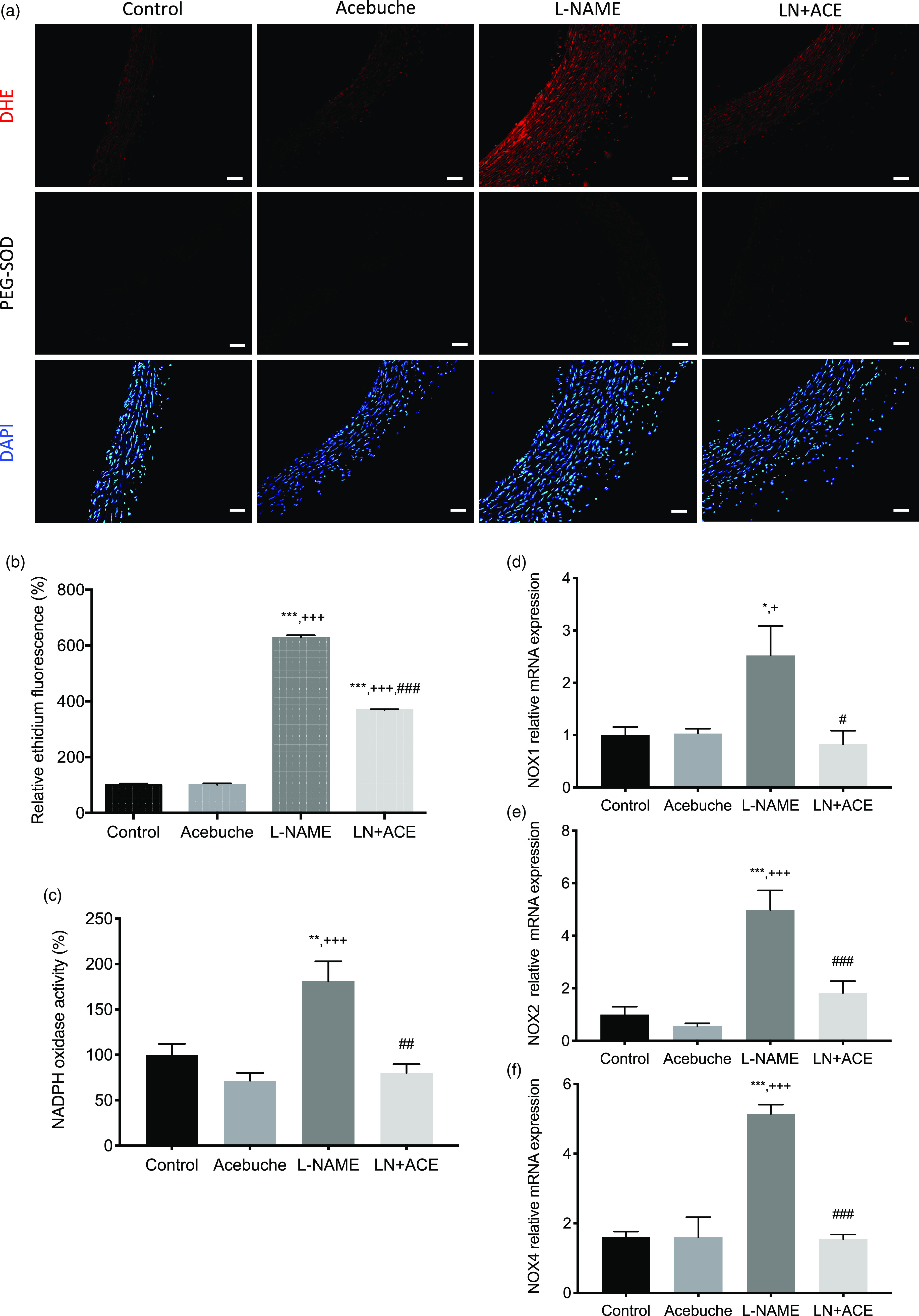
(a) Dihydroethidium (DHE) labelling (red colour) for superoxide anion production and 4´,6-diamidino-2-phenylindole (DAPI, blue colour) nuclei staining in the different experimental groups. Middle line photos in the panel represent the effects of preincubation with polyethylene glycol-conjugated superoxide dismutase (PEG-SOD); (b) superoxide anion quantification (% relative to DAPI) relative to that of Control; (c) NADPH oxidase enzyme activity; (d)–(f) gene expression of NOX1, NOX2 and NOX4, respectively, in aorta homogenates of rats from the different experimental groups. Values are expressed as mean values with their standard errors of the mean of at least four animals per group. *P < 0·05, **P < 0·01, ***P < 0·001 *v*. Control group; ^+^ *P* < 0·05, ^+++^ *P* < 0·001 *v*. Acebuche group; ^#^ *P* < 0·05, ^##^ *P* < 0·01, ^###^ *P* < 0·001 *v*. L-NAME group. Scale bar: 50 µm.

Quantification of DHE-stained images resulted in a 6-fold augmentation of superoxide anion (O_2_^•^-) release in the aorta of rats exposed to L-NAME, when compared with the Control and Acebuche groups (Fig. 5(a) and (b)). Dietary supplementation with ACE oil in hypertensive animals attenuated this L-NAME-dependent overproduction of superoxide. DHE staining disappeared when aortic ring preparations were preincubated with polyethylene glycol-conjugated SOD (Fig. 5(a)), thus confirming the presence of O_2_^•^- and the specificity of the staining in our experimental conditions.

Besides O_2_^•^- quantification by DHE staining *in situ*, the activity of NADPH oxidase was also increased in aorta homogenates from L-NAME-treated rats compared with the Control group, which was prevented by ACE oil-enriched diet (Fig. 5(c)). These results also paralleled the gene expression pattern observed for the NADPH oxidase isoforms NOX1, 2 and 4 (Fig. 5(d)–(f)).

### Antioxidant enzyme activity (peripheral blood) and expression (aorta)

**Fig. 6.**
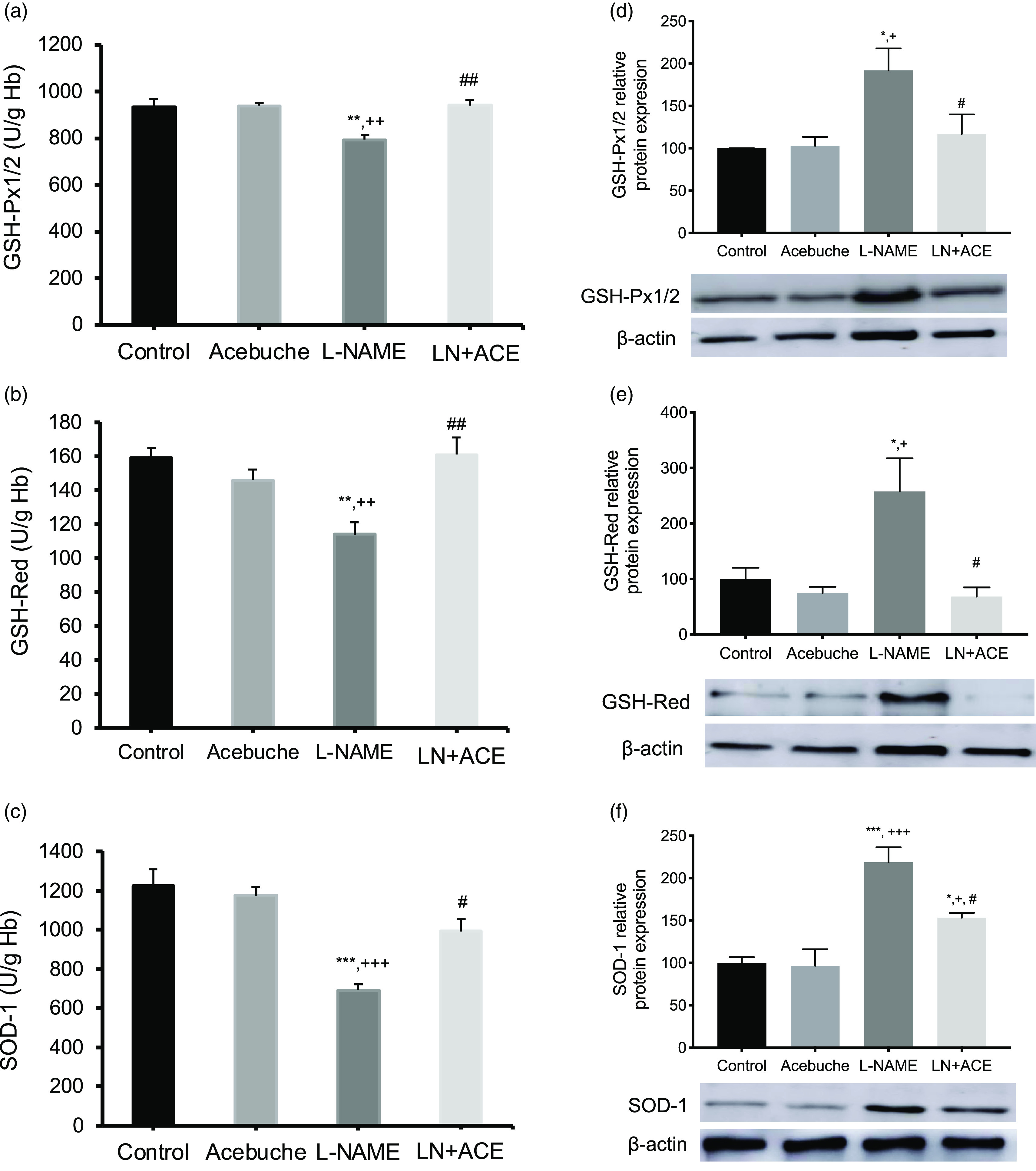
(a) Glutathione peroxidase (GSH-Px1/2); (b) glutathione reductase (GSH-Red); and (c) superoxide dismutase (SOD-1) activities in peripheral blood; (d)–(e) protein expression of antioxidant enzymes depicted in (a)–(c), respectively, in aorta homogenates of rats from the different experimental groups. Values are expressed as mean values with their standard errors of the mean of six animals per group. *P < 0·05, **P < 0·01, ***P < 0·001 *v*. Control group; ^+^ *P* < 0·05, ^++^ *P* < 0·01, ^+++^ *P* < 0·001 *v*. Acebuche group; ^#^ *P* < 0·05, ^##^ *P* < 0·01 *v*. L-NAME group.

As shown in Fig. 6(a)–(c), a significant decrease in the enzymatic activity of GSH-Px1/2 (11 %), GSH-Red (31 %) and SOD-1 (43 %) was found in peripheral blood of hypertensive animals, which was reversed by ACE oil-enriched diet. On the other hand, additional experiments in aorta homogenates showed a hypertension-dependent increase in the protein expression of the same enzymes; this alteration was also corrected with the daily consumption of ACE oil (Fig. 6(d)–(f)). Again, no modifications were observed between the Control and Acebuche groups, which behaved similarly in this study with the sole exception of the plasma levels of HDL-cholesterol (Table 2).

## Discussion

The health benefits of EVOO in CVD have been extensively reported. The effects are mainly attributed to its high content in triterpenic and phenolic compounds that attenuate CVD prevalence, such as hypercholesterolaemia, hypertension and atherosclerosis^(42)^. These bioactive compounds, together with tocopherols and sterols, can contribute to vasodilator, antioxidant and anti-inflammatory properties of this oil^(13,14,19,43)^.

*Olea europaea*, L. var. *sylvestris*, often known as ACE or wild olive tree, is a naturally occurring variety that is widely distributed in certain areas of Mediterranean countries. Despite the importance of forest biodiversity and the potential interest in preserving the primitive lineages of olive trees, they are still far from being characterised, and very little is known about the health properties of ACE-derived products, including ACE oil. Recent analyses of the chemical composition of ACE oil carried out in our lab have revealed the presence of a high proportion of minor constituents such as tocopherols, sterols, triterpene acids and alcohols, and secoiridoid polyphenols^(27)^. Interestingly, our pioneering studies have also demonstrated the antioxidant and retinoprotective capacity of an ACE oil-enriched diet in the eye of hypertensive mice^(27)^.

In the present study, we have evaluated the health benefits of ACE oil on specific functional and morphological changes involved in hypertension-associated vascular damage. L-NAME is a well-established compound to study the pathophysiology, therapeutics and organ damage associated with primary hypertension^(44)^. Chronic exposure to this competitive inhibitor of eNOS reduces NO formation and leads to vasoconstriction, endothelial dysfunction and hypertrophic vessels^(45)^, which eventually results in sustained elevation of BP. Our results suggest that the daily consumption of ACE oil can counteract the harmful effects of L-NAME by exerting vasodilator, antihypertensive, antihypertrophic and antifibrotic effects. In agreement with previous reports, the administration of L-NAME did not affect the animals’ body weight^(46)^. On the other hand, as expected, L-NAME induced a significant elevation of systolic and diastolic blood pressure values^(40,45)^, which was attenuated after dietary supplementation with ACE oil, interestingly. Recent studies using EVOO demonstrated a reduction of BP in spontaneously hypertensive rats (SHR)^(47)^. Subsequently, a decrease in systolic blood pressure was also observed in SHR after enriching a VOO with phenolic compounds^(48)^, indicating an association between olive polyphenols and positive BP outcomes^(49)^. Therefore, the hypotensive effect found in ACE oil might be related to the high content of secoiridoids, one of the minor components that is present in this product^(27)^.

In the present work, no differences in total cholesterol, lipids and phospholipids levels in plasma were found among different animal groups. These results differ from those reported by Belarbi et al., who observed that oleaster oil positively modulated plasma lipids in humans, with a reduction in total cholesterol and TAG^(22)^. In agreement with this study, our results did show an increase in HDL-cholesterol accompanied by reductions of TAG and LDL-cholesterol in hypertensive animals fed with ACE oil.

Our model of hypertension secondary to NO depletion presented a very significant increase in plasma urea concentration that could be abolished by ACE oil. Such an observation supports the notion that renal function might benefit from regular intake of wild olive oil. In this regard, several studies have described a recovery in rats with induced nephrotoxicity after treatment with VOO or EVOO^(50,51)^.

Our results were accompanied by relevant alterations of vascular reactivity in hypertensive aortic rings, with an increase in the vasoconstrictor response to phenylephrine and a decrease in the ACh/endothelium-dependent vasodilation capacity. This altered response to vasoactive mediators could be improved after simultaneous administration of ACE oil to L-NAME-treated animals. As expected, acute preincubation of aorta tissue with L-NAME reduced ACh-mediated vasodilation in all experimental groups, with the lowest response being observed in the group previously subjected to chronic, 12-week NO depletion (L-NAME group). Under these conditions, ACE oil managed to raise E_max_ values in hypertensive animals to those obtained in normotensive (Control and Acebuche) groups. Regarding endothelium-independent vasorelaxation with sodium nitroprusside, no particularly relevant changes were observed between the four experimental groups. All these findings suggest that the hypotensive effect of ACE oil might be exerted, at least in part, in an endothelium-dependent manner via improving NO bioavailability.

Improvement of endothelial function by inhibition and/or scavenging of radical oxygen species has been found after administration of VOO^(52)^ and VOO enriched with phenolic compounds^(48)^ in SHR. Interestingly, the administration of a high polyphenol VOO-enriched (15 % w/w) diet for 20 weeks was able to restore endothelial function in mesenteric arteries from apolipoprotein-E-deficient (ApoE KO) mice, suggesting a major role of polyphenols in counteracting the progression of atherosclerotic plaques^(53)^. In addition, the beneficial effect of Mediterranean diet on endothelial function has recently been reported in patients with CHD^(54)^. Triterpenic compounds such as oleanolic acid and maslinic acid, as well as triterpene alcohols erythrodiol and uvaol, elicited a high vasorelaxation response in aortic rings from hypertensive animals^(14)^, and improvement of endothelial function in healthy humans^(15)^. Furthermore, our results are in agreement with data showing that chronic administration of oleuropein-enriched (15 % w/w) olive leaf extract to spontaneously hypertensive rats re-established ACh-induced vasodilation in a NO-dependent manner^(55)^.

The underlying mechanisms responsible for antihypertensive and vasodilator effects of triterpenic compounds include up-regulation and increased activity of eNOS, thus leading to a rise in NO release and favouring vasorelaxation^(56)^. In this sense, the results of the present study showed that altered eNOS activation/expression and NO bioavailability in the aortas of rats with L-NAME-induced hypertension were alleviated with dietary ACE oil. The high content of triterpene and secoiridoid compounds found in ACE oil^(27)^ might account for the improvement of vascular function observed in our experimental design, as has also been reported in rat mesenteric arteries^(57)^. In fact, we have recently described that ACE oil also induced NO release in the retina of hypertensive mice by increasing eNOS activation, estimated as the ratio of eNOS phosphorylation at Ser^1177^/Thr^495^ ^(27)^.

To evaluate the effect of ACE oil on blood vessel architecture, morphometric and fibrosis studies were performed in aorta sections from all animal groups. Our results showed the presence of vascular remodelling in hypertensive animals that could be reduced in the group receiving the oil together with L-NAME. Moreover, the abnormally elevated content of collagen fibres in aortas from NO-depleted rats, as well as the elevated expression of the profibrotic cytokine TGF-*β*1 at this level, was normalised by the ACE oil-enriched diet. Alterations in TGF-*β*1 activity have been described in different pathologies including atherosclerosis, cancer, hypertension or cardiomyopathies^(58)^. Indeed, this cytokine can act as a positive or negative regulator of fibrotic and inflammatory processes, cell proliferation and migration. In the present study, the process of vascular fibrosis was improved when ACE oil was administered to hypertensive rats on a daily basis. Our findings are in accordance with studies describing a reduction in aortic media thickness of SHR after using oils enriched with oleic acid^(59)^. In addition, previous reports evidenced that an olive oil concentrated in triterpenic acids decreased collagen deposition and TGF-*β*1 expression in aortas from SHR^(60)^. Taken together, all these findings suggest that ACE oil’s antihypertrophic and antifibrotic effects might be related to its hypotensive potential and could be attributable to the high content of oleic acid and triterpene compounds found in this oil. Interestingly, previous studies in resistance arteries of atherosclerotic mice concluded that polyphenols did not provide any additional benefit in vascular wall structure, mechanics or myogenic response, to those traditionally attributable to VOO^(53)^.

The interplay among oxidative stress, AH and fibrosis is well established. Oxidative stress is partly produced by the interaction between the enzyme NADPH oxidase and TGF-*β*1; this is one of the mechanisms involved in the synthesis of extracellular matrix and accumulation of collagen fibres in the intima layer during the process of vascular remodelling^(61)^. In our study, we found an increase in superoxide anion (O_2_^•^-) content in the aorta of hypertensive rats that was ameliorated following regular intake of ACE oil for 12 weeks. This observation was paralleled by additional experiments on the activity and expression of NADPH oxidase enzyme. Our results in this regard are in agreement with the decline in O_2_^•^- production observed in mesenteric arteries of atherosclerotic mice following 20-week supplementation with VOO^(53)^. Previous studies in our lab have also demonstrated a significant blockade of O_2_^•^- release in retinas from hypertensive mice treated with ACE oil, an observation that correlated with down-regulation of NADPH oxidase^(27)^. With the present study, we confirm the antioxidant capacity of ACE oil in the hypertensive context by inhibiting NADPH oxidase-dependent reactive oxygen species production.

Oleocanthal, one of the major phenolic secoiridoids present in olive oils, produced a decrease of reactive oxygen species in lipopolysaccharide-activated murine peritoneal macrophages^(62)^. Therefore, the inhibition of NADPH oxidase activity and O_2_^•^- production achieved after ACE oil consumption might be attributed to the relatively high content of secoiridoids present in this oil^(27)^. Otherwise, the elevated SOD-1 activity (responsible for enzymatic O_2_^•^- neutralisation) observed in the peripheral blood of ACE oil-fed hypertensive rats might also account for the decline in superoxide content demonstrated in these animals. Additional increases in the systemic activity of antioxidant enzymes GSH-Px1/2 and GSH-Red after administration of ACE oil might contribute to its antioxidant capacity by eliminating excess H_2_O_2_ and by replenishing reduced glutathione (GSH), the latter being one of the main components of the non-enzymatic antioxidant defence system. However, the relative protein content of antioxidant enzymes in the aorta was clearly higher in hypertensive animals, an alteration that was corrected by dietary ACE oil. These results indicate that the activity and expression of antioxidant enzymes are tissue dependent.

In conclusion, the results obtained in the present study demonstrate that regular consumption of ACE oil triggers vasodilator, antihypertensive, antihypertrophic and antifibrotic effects in L-NAME-induced hypertensive rats. Therefore, the incorporation of ACE oil to the diet may represent a useful tool against AH and vascular remodelling, which could help reduce risk factors for CVD. We must note, however, a limitation of our study in that histological and biochemical improvements have been reported in aortic tissue from LN + ACE-treated rats; further studies on resistant vessels (e.g. mesenteric arteries) will probably help elucidate those vascular molecular mechanisms involved.
